# Cross-Cultural Adaptation and Measurement Properties of the Indonesian Kujala Score for Anterior Cruciate Ligament Tear Patients

**DOI:** 10.5704/MOJ.2207.003

**Published:** 2022-07

**Authors:** R Deviandri, V Yuliana, AP Kautsar

**Affiliations:** 1 Department of Orthopedics, University Medical Center Groningen, Groningen, The Netherlands; 2 Faculty of Medicine, Universitas Riau, Pekanbaru, Indonesia; 3 Department of Surgery, Arifin Achmad Hospital, Pekanbaru, Indonesia; 4 Department of Sport Health, FIT Sport and Rehabilitation Centre, Pekanbaru, Indonesia; 5 Faculty of Pharmacy, Universitas Padjadjaran, Bandung, Indonesia; 6 Department of Health Sciences, University Medical Center Groningen, Groningen, The Netherland

**Keywords:** translation, validity, reliability, Kujala-Indonesian version, ACL injury

## Abstract

**Introduction:**

There is an overwhelming need for worldwide applicable subjective grading systems for patients' anterior cruciate ligament (ACL) tear. The scoring system should be validated for their use in advance related measurements. For the Indonesian language speaking population, only the Kujala Patellofemoral Score (KPS) questionnaire has been translated and validated into the Indonesian language for diseases related to knee pain, but none for ACL tears. The present study aims at cross-cultural adaption to measure the validity and reliability of the Kujala patellofemoral score Indonesian version (KPS-I) specifically for ACL tear patients.

**Material and methods:**

The responses of 106 ACL tear patients on two questionnaires comprising the KPS-I and Short Form (SF)–36 were examined by determining the validity and reliability. We conducted the validity construct and content, so the reliability was evaluated by test-retest reliability, internal consistency and measurement error. In addition, the research utilised the Bland and Altman method to explore absolute agreement.

**Results:**

The construct and content validity were good, where all hypotheses were confirmed, and the floor or ceiling effect did not occur. The reliability proved excellent between test and retest (ICC=0.99). An internal consistency showed a good Cronbach α of 0.86. The standard error of measurement (SEM), minimal detectable change at the individual (MDCind), and minimal detectable change at the group (MDCgrp) were determined to be 2.1, 5.8, and 0.7, respectively. The application of the Bland and Altman plot method revealed no bias in this study.

**Conclusion:**

The validation procedure shows that the KPS-I is a good evaluation instrument for Indonesian patients with ACL tear. However, it is suggested that this score be used for follow-up of patients after ACL reconstruction procedure, especially with anterior knee pain related to the original objective of the Kujala score.

## Introduction

ACL tear rate has increased gradually in the recent decade. The annual global age and sex-adjusted ACL tears incidence were 74.6 per 100,000 person-years^1^. To recover the function of the knee, some treatment must be conducted, such as ACL reconstruction (ACLR) surgery. The treatment also has an impact on the patient to do sports again^2,3^. To evaluate and achieve this goal, patient-reported outcome measures (PROMs) or questionnaires were needed.

There is a great need for applicable subjective grading systems for patients' anterior cruciate ligament (ACL) tear. The scoring system should be validated for their use in advance related measurements to be performed. For Indonesian speaking patients, only the Kujala Patellofemoral Score (KPS) questionnaire has been translated and validated into the Indonesian language for knee pain-related disease, but not for ACL tear cases.

The questionnaire is an essential tool in orthopaedic surgery to evaluate the impact of any surgical procedure on patients' daily lives. Several health-related qualities of life (HR-QoL) questionnaires have been developed; some are generic, others are specific, or are joint-specific in the case of musculoskeletal issues. The Kujala Patellofemoral Score (KPS) is frequently used PROMs to measure knee-specific disease^4^. The tool is also intended to determine the patient's functional status with patellofemoral pain^5^. They have been translated and used in the Indonesian language^6^. However, to use this KPS-I for ACL tear patients, this questionnaire should be validated. Growing body of evidence showed a positive correlation between KPS and some ACL-specific PROMs. Celik *et al* found an excellent correlation between KPS and International Knee Documentation Committee (IKDC) for ACL tear patients^7^. Furthermore, Mustamsir *et al* explained that KPS could evaluate ACL tear patients followed by anterior knee pain6. Nevertheless, the literature relating to the reliability and validity of KPS-I for patients with ACL tears is still unavailable.

Thus, the purpose of the study was to adopt a cross-cultural approach, and assess the validity and reliability of the KPS-I questionnaire in patients with an ACL tear.

## Materials and Methods

Informed consent from patients was obtained for participation in this research as per the principles of the Helsinki declaration. We obtained approval for the study from the institutional review board. We used KPS-I and SF-36 questionnaires for this study. The KPS-I is a disease-specific questionnaire of anterior knee pain. It consists of six activities associated explicitly with anterior knee pain. The 13 questions' KPS-I has a total score ranging from 0 to the maximum of 1005. The SF-36 generic questionnaire consists of eight sections, and the scores range from 0 to 100. The better health condition is reflected by the higher scores^8^.

KPS-I was prepared originally from the literature6, then distributed to ten patients with ACL tears during follow-up in the outpatient knee clinic of one hospital in Indonesia. ACL reconstruction. The researcher documented any difficulties experienced among patients during the completing of the questionnaire. The documentations were reviewed, and some cultural adaptation was made during the preparation. The word "squatting" in KPS-I was extended to "squat during prayer," which is quite common among the Indonesian population. The word "atrophy" was not familiar to some patients, so we added the explanation "decreasing the size of thigh muscle" to make this statement clearer.

Between April until September 2020, a total of 253 randomly selected participants were recruited to contribute to the research. All subjects were Indonesian patients who underwent reconstruction for their ACL tears between January 2015 until March 2020 at one of the hospitals in Indonesia. We included patients following ACL reconstruction for ACL tears, either isolated or multi-ligament injury, either acute or chronic, with or without concomitant meniscus injury. Patients were then sent information about this study and with two sets of questionnaires (Parts 1 and 2) by email. Part 1 was sent in the first week and included the KPS-I and SF-36. Part 2 was sent in the second week with only the KPS-I. We instructed patients to complete and submit each set of questionnaires and forwarded them to the researcher's address immediately. The patients were provided a global rating of change (GRC) question to be asked at the beginning of Part 2 about the subject's condition between the interval of the two times of completing the questionnaire. This step is needed to establish that the health and knee function persist stably between the recurrent examinations. We included patients with unchanging knee functions within the time interval of the test-retest analysis. In contrast, patients who re-sent each of the questionnaires on the same day or more than one month apart were excluded.

The KPS-I construct validity was tested by correlating it to the SF-36's physical domains (physical component summary, physical function, and physical role functioning). The content validity was evaluated by the ceiling and floor effects' distribution and occurrence. It means that the ceiling and floor effects were considered to be present if more than 15% of participants reached the lowest or highest possible score^9^.

Predefined hypotheses about the relationship's size between the KPS-I and subscales of the SF-36 were formulated according to the COSMIN guidelines^10^. The KPS-I correlates with physical functioning better than social and emotional aspects. Therefore, stronger correlations were expected between the KPS-I and physical component summary (PCS) subscales of the SF-36. Then, lower correlations were expected between the KPS-I and the mental component summary (MCS) subscale of SF-36. The study of Apivatgaroon *et al*^11^ showed a correlation of 0.59 between the Thai version-KPS and the PCS subscale of SF-36 and 0.41 with the MCS subscale of SF-36. Then, it showed a correlation of 0.52 between the Thai version-KPS and the role physical functioning subscale of SF-36.

On the other hand, the correlation between the Thai version-KPS and either social functioning or emotional role functioning subscale of SF-36 showed 0.25 and 0.47, respectively. Based on the correlations found in that study, it was hypothesised to find correlations of 0.5 or higher between the KPS-I and the PCS, physical functioning, and physical role functioning subscale of SF-36. Despite this, it was hypothesised to find correlations of 0.5 or lower between the KPS-I and the MCS, social functioning, and emotional role functioning subscale of SF-36. All predefined hypotheses are shown in Table I.

**Table I:** Predefined hypotheses

1	Correlations ≥ 0.5 between the KPS-I and the physical functioning
2	Correlations ≥ 0.5 between the KPS-I and the physical role functioning
3	Correlations ≤ 0.5 between the KPS-I and the emotional role functioning
4	Correlations ≤ 0.5 between the KPS-I and the social functioning
5	Correlations ≥ 0.5 between the KPS-I and the PCS
6	Correlations ≤ 0.5 between the KPS-I and the MCS
7	Correlations between KPS-I and PCS subscale of SF-36 stronger than correlations between KPS-I and MCS subscale of SF-36

We conducted the reliability test represented by internal consistency, test-retest reliability, and measurement error to identify individuals according to the COSMIN guidelines^10,12^. In addition, to explore the absolute agreement, the Bland and Altman technique was used. The result reflected the amount of agreement in recurring measurements^13^.

The characteristics of the study population and scores on the questionnaires were described using means and standard deviations (SD), or frequencies and percentages. Pearson correlation coefficients were calculated between the scores on the KPS-I and the other questionnaires to determine to construct validity. The values less than 0.10, 0.10-0.39, 0.400.69, 0.70-0.89, and greater than 0.90 indicate a negligible, weak, moderate, strong, and robust correlation^14^.

To determine internal consistency, Cronbach-α was calculated^15^. The ranges value 0.70-0.95 indicated a decent internal consistency^9^. The intraclass correlation coefficient (ICC) between test and retest KPS-I scores was calculated to assess the test-retest reliability^10^. Values less than 0.5, 0.50.75, 0.75-0.9, and greater than 0.90 indicate poor, moderate, reasonable, and excellent reliability, respectively^16^. Measurement error was assessed with SEM and MDC. We calculated SEM by multiplying the pooled standard deviation by √(1-r), where r is the ICC^17^. We calculated MDCind using the formula 1.96 × SEM × √2 and at the group level, MDCgrp was divided by √n^9^.

Absolute reliability was assessed using Altman-Bland plots; when 0 was in the 95% confidence interval (CI) of the mean difference between the test and retest score, no consistent bias is present. Then, we calculated the 95% limits of agreement (LoA) using the formula mean difference ± 1.96 × SDdiff (SDdiff = SD of the mean difference between the first and second administration of the KPS-I)^13^.

The data analyses and interpretation were performed using the SPSS Statistics version 26.0 (IBM) by significance level at 5%.

## Results

**Fig 1:**
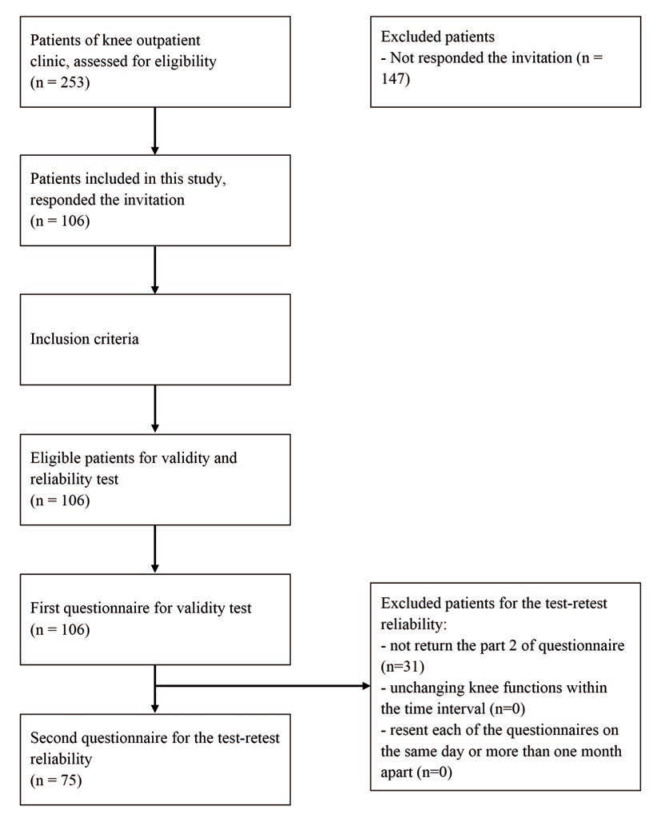
The framework of the study process

A total of the 253 patients were recruited to participate; 106 patients (42%) responded and returned the first mailing of questionnaires. Of 106 patients, 75 patients (70.8%) filled in and returned both Part 1 and Part 2 of the questionnaires. The remaining 31 patients (29.2%) returned only Part 1. No patients reported a changed function of the operated knee when they filled out Part 2 of the questionnaire. Hence, the material part of the analysis was conducted on 106 patients' data and test-retest examination on 75 patients' data. Fig. 1 depicts the framework of this study. The demographic characteristic of patients and the main results of the questionnaire are shown in Tables II and III.

**Table II:** Demographic Patient Characteristics (n=106)

Characteristic	Value
Age, mean ± SD Age group, n (%)	30.49 ± 9.18
Sex, n (%)	
Male	75 (70.8)
Female	31 (29.2)
Affected side, n (%)	
Right	60 (56.6)
Left	46 (43.4)
Both	0
Diagnose, n (%)	
Isolated ACL	103 (97.2)
Multi ligament injury	3 (2.8)
Occupation, n (%)	
Athlete	14 (13.2)
Non-athlete	92 (86.8)
Activity at an injury, n (%)	
ADL	11 (10.3)
Sport	73 (68.9)
Traffic	20 (18.9)
Work	2 (1.9)

**Table III:** Descriptive Statistics for the PROMs Statistics

Measure	Mean ± SD
KPS-I	81.57 ± 14.92
SF-36 Physical Functioning	68.54 ± 26.63
SF-36 Physical Role Functioning	35.85 ± 41.98
SF-36 Bodily Pain	72.14 ± 24.22
SF-36 General Health Perceptions	67.53 ± 18.30
SF-36 Vitality	65.14 ± 20.65
SF-36 Social Function	68.63 ± 21.15
SF-36 Emotional Role Functioning	46.54 ± 45.47
SF-36 Mental Health	69.92 ± 22.20
SF-36 PCS	43.6 ± 9.7
SF-36 MCS	35.2 ± 16.6

The validity test showed that the KPS-I was strongly related to the physical domains of the SF-36 (physical functionality, role physical functioning) than to its cognitive domains (emotional role functioning, social functioning) as mentioned in (Table IV) (0.52, 0.50, 0.45, and 0.33, respectively). Furthermore, the KPS-I was strongly related to the PCS than the MCS subscale of SF-36 (0.60 and 0.50, respectively).

**Table IV:** Correlations Between KPS-I and the Domains of the SF-36

SF-36	KPS-I
Physical functioning	0.52
Physical role functioning	0.50
Bodily pain	0.58
General health perception	0.42
Vitality	0.43
Social function	0.33
Emotional role functioning	0.45
Mental health	0.45
PCS	0.60
MCS	0.50

The study confirmed all predefined hypotheses on the extent of associations between the KPS-I and the SF-36. The floor and ceiling effects were less than 15% (12%).

From the reliability test, the Cronbach-α showed 0.86 and 0.82 in the test and retest assessment, respectively. The ICC of KPS-I had a value of 0.99 (p< 0.001), with the 95% CI ranging from 0.99 to 1.0.

**Fig 2:**
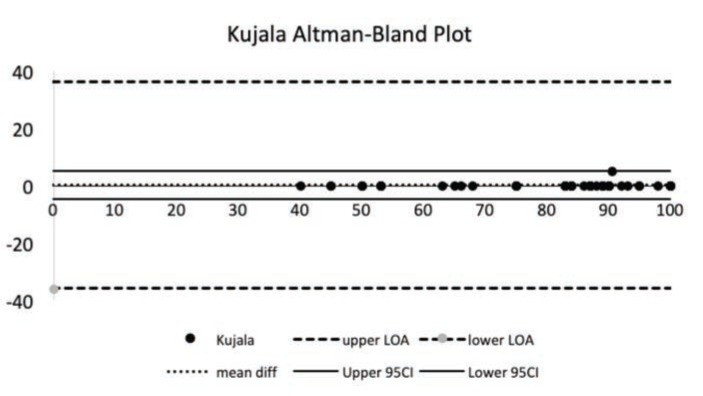
The Altman-Bland plot showed that 95% CI was still in the range of the limits of agreement and showed no bias.

The measurement error test found that the SEM, MDCind, and MDCgr were determined to be 1.57, 4.34, and 0,7, respectively. The Altman-Bland approach revealed a mean difference of the two assessments of 0.1 with 95% CI= -4.95.1, 95% LoA= -30.73-30.86 (Figure 2). Nonconsistent bias was presented because the value of 0 was in the 95% CI of the mean difference between the test and retest score.

## Discussion

This study aimed to get the validity and reliability of the KPS-I questionnaire in patients with an ACL tear. With the increasing number of ACL tear cases, international questionnaires for outcomes assessment are needed. The Kujala questionnaire is frequently used in anterior knee pain cases, and being a knee-specific questionnaire, it could also be used in other knee diseases. From this study, we found that KPS-I was valid and reliable for ACL tear cases.

The construct validity was assessed by outlining seven hypotheses about the magnitude of the association between the KPS-I score and SF-36. All hypotheses confirmed are the indication of good construct validity. The KPS-I score showed a stronger correlation with the physical component summary, the physical functioning, and the physical role functioning of the SF-36 (p<0.001). In this case, KPS-I can be indicated as a tool of examination in function and sports activities since the validity showed a good result. Although KPS-I also showed a moderate correlation to the MCS subscale of the SF-36, this correlation was weaker than its PCS subscale.

Further affirming the level of validity of the KPS-I, acceptable levels of ceiling and floor effects were found of less than 15% (12%). These are higher than those reported in previous studies, such as the Thai version^10^ that did not find ceiling and floor effects. The ceiling and floor effect in this study may be due to the peculiarity of the sample selected (age and type of sports activity of the sample).

The KPS-I test-retest reliability result showed outstanding reliability (ICC 0.99). The result can be compared (Table V) with the Portuguese version (ICC 0.86)18, the German version (ICC 0.93)^19^, the Dutch version (ICC 0.98)^20^, the Persian version (ICC 0.96)^21^, and the Thai version (ICC 0.91)11. The internal consistency showed a good result (Cronbach-α 0.86). The result can be compared with the Thai version (0.95)^11^, Portuguese version (0.80)^18^, the German version (0.87)^19^, the Dutch version (0.78)^20^, the Persian version (0.81)^21^, the English version (0.83)^22^, the Chinese version (> 0.70)^23^, the Spanish version (0.77)^24^ and the Turkish version (0.84)^25^.

**Table V:** Psychometric Properties of Kujala Score Including KPS-I

Study	Language Version	KPS-I Retest Reliability ICC	KPS-I Cronbach Alpha
Present study	Indonesian	0.99	0.86, 0.82
Gil-Gamez *et al*^24^	Spanish	0.99	0.77, 0.80
Ummels *et al*^20^	Dutch	0.98	0.78, 0.80
Apivatgaroon *et al*^11^	Thai	0.91	0.95
Dammerer *et al*^19^	German	0.93	0.87
Kuru *et al*^25^	Turkish	NA	0.84
Negahban *et al*^21^	Persian	0.96	0.81
Ittenbach *et al*^22^	English	NA	0.83
Cheung *et al*^23^	Chinese	0.97	> 0.70
Da Cunha *et al*^18^	Brazilian, Portuguese	0.86	0.80

The time interval used between the test-retest reliability is essential. In general, the time interval between repeat administration for a patient-reported outcome measurement (PROM) should be relatively brief (three to seven days) when the condition being measured is expected to change rapidly^7^. Furthermore, Terwee *et al* stated that the time interval between the test-retest reliability should be long enough to prevent recall, though short enough to ensure that clinical change has not occurred. Often, one or two weeks will be appropriate^9^. In this study, we used a one-week time interval to ensure the individual's condition was stable between the test-retest reliability, as used in some studies for ACL tear patients^7^.

The SEM, MDCind, and MDCgr were 1.57, 4.34, and 0.7, respectively. Small values of group level's MDC showed that the KPS-I is adequate compared to groups. The MDC's low values are necessary to detect alteration. The value was comparable to the presented value's English version (SEM of 0.82 to 3.0)^22^, or the Dutch version, which presented an SEM and MDC of 3.9 and 11.0, respectively^20^. The MIC should be determined whether there are any differences between the group and prove clinically significant^9^. For the prospective research evaluative purposes, every MIC of the KPS-I subscale should be determined.

The Altman-Bland plots showed a satisfactory agreement, reporting only a slight variance between the averages. It was shown that no consistent bias was presented because the value of zero was in the 95% CI of the mean difference between the test and retest score.

There are some limitations in this study. First, the response rate is relatively low (42%). We supposed this is because some patients were unfamiliar with email and an online questionnaire, typically among the Indonesian population. In future studies, we suggest trying another technique to increase this rate, such as by direct contact in the outpatient clinic. However, at least 100 patients were needed for the validity test, and 50 patients were needed to conduct test-retest reliability based on COSMIN guidelines^10^.

Second, the patients were examined by themselves. We could not control treatments of the patients during the test-retest, which may have altered their symptoms. Thus, we asked the patients to explain their initial symptoms and conducted the test again. However, this study is the first to evaluate the validity and reliability of KPS-I among Indonesian patients with ACL. Third, some psychometric properties have not been measured. The capability of this KPS-I to detect change over time has not been examined. The responsiveness of KPS-I should also be determined in future research.

## Conclusion

The KPS-I is a valid and reliable instrument to assess the functional outcome of ACL tear cases for the Indonesian-speaking population. However, it is suggested to use this score to follow-up the patient after the ACL reconstruction procedure, especially with anterior knee pain related to the Kujala score's original purpose. In addition, further study is required into the minimal important change and responsiveness of Kujala score for ACL tear patients.
